# “To patent or not to patent? the case of Novartis’ cancer drug Glivec in India”

**DOI:** 10.1186/1744-8603-10-3

**Published:** 2014-01-06

**Authors:** Ravinder Gabble, Jillian Clare Kohler

**Affiliations:** 1 Dalla Lana School of Public Health, University of Toronto, 155 College Street, 6th floor, Toronto, Ontario M5T 3 M7, Canada; 2 Associate Professor and Director Global Health, Leslie Dan Faculty of Pharmacy, & Munk School of Global Affairs, University of Toronto, 144 College Street, Toronto, Ontario M5S 3 M2, Canada

**Keywords:** India, Novartis, Glivec, Patents, Generic drugs, TRIPS agreement, Drug industry, Pharmaceuticals

## Abstract

**Background:**

Glivec (imatinib mesylate), produced by the pharmaceutical company Novartis, is prescribed in the case of Chronic Myeloid Leukemia, one of the most common blood cancers in eastern countries. After more than a decade of legal battles surrounding its patentability, the Supreme Court of India gave its final decision on April 1st of 2013, rejecting the appeal of the Swiss giant drug manufacturer. In 2006, the Indian Patent Office first refused Glivec’s patent under Section 3(d) of the Indian Patent Act arguing that it was only a modified version of an existing drug, Imatinib, and therefore that the drug was not innovative. Novartis replied filing legal challenges against the Indian government but the final verdict in April of 2013 ends the battle. Indeed, the Supreme Court stated that even if the bioavailability of the drug was improved, it did not demonstrate enhanced efficacy and that Glivec was not patentable.

**Methods:**

The research primarily focused on journal, newspaper and magazine articles relevant to the time frame of the lawsuit (from 1994 to 2013) as well as news searches through Google, Factiva, ProQuest, PubMed, and YouTube where press articles from court verdicts were obtained by using the following keywords: “India”, “Novartis”, “Glivec”, “Patent”, “Novartis Case”, and “Supreme Court of India”. The data sources were interpreted and analyzed according to the authors’ own prior knowledge and understanding of the exigencies of the TRIPS Agreement.

**Results:**

This case illuminates how India is interpreting international law to fit domestic public health needs.

**Conclusions:**

The Novartis case arguably sets an important precedent for the global pharmaceutical industry and ideally will help improve access to lifesaving medicines in the developing world by demanding that patient health needs supersede commercial interests. The Supreme Court of India’s decision may affect the interpretation of the article of the TRIPS Agreement, which states members shall be free to determine the appropriate method of implementing the provisions of this Agreement within their own legal system and practice.

## Background

India’s pharmaceutical industry is considered as the 3rd largest in the world in terms of volume and the 14th in terms of its value. With China, Brazil and Russia, it led a group of seventeen high-growth pharmaceuticals markets also called “pharmerging countries” which are expected to contribute to nearly 50% of the annual pharmaceutical market growth in 2013 [1]. According to the research firm IMS Health, sales in those emerging markets are predicted to reach 30% of global pharmaceutical spending in 2016, compared to 20% in 2011. India’s robust pharmaceutical industry was estimated at over USD $10-billion in 2010 [2]. By 2020, pharmaceutical sales in India are predicted to grow to as much as USD $74 billion – over six times than what they were in 2010 [3]. But, despite its thriving pharmaceutical market, improving its population’s access to medicines is a key concern for a country that has nearly “70% of its population living on less than USD $2 per day” [4] and only 5% with access to private health insurance [5]. Generic pharmaceutical manufacturers dominate the Indian pharmaceutical market, accounting for up to 90% of product sales [6]. According to Yusuf Hamied, chairman of the Indian pharmaceutical company CIPLA, “India boasts more drug-manufacturing facilities that have been approved by the U.S. Food and Drug Administration than any other country outside of the United States” [7].

Given its capacity to produce large quantities of drugs at cheap, affordable prices, India is known to many as the “Pharmacy of the Developing World” as it has become a leading supplier of generic medicines to many developing countries [8]. For example, India’s production of HIV/AIDS medications has helped lower the cost of treatment (a combination of stavudine, lamivudine, and nevirapine) dramatically from as much as USD $10,000 per year in 2000 to USD $150 per year today [8]. India now supplies 80% of the 6 million people receiving treatment for HIV/AIDS in the developing world today [9]. India also stands as “the second leading provider of medicines distributed by UNICEF in the developing world”. [10] Since 2005, India has been obliged pursuant to the Trade Related Aspects of Intellectual Property Rights (TRIPS) Agreement to put into place revised patent laws in line with global standards. This has in turn presented the country with a number of legal cases that have had the potential to threaten its standing as a major supplier of low-cost generic medicines globally. In our paper, we examine the significant rejection, on April 1st, 2013, by the Supreme Court in India of an appeal by the giant Swiss pharmaceutical company Novartis to patent a modified version of its cancer drug, Glivec (imatinib mesylate).

### India and the TRIPS agreement

For well over 30 years, the Indian government did not allow product patents for pharmaceutical inventions, paving the way for Indian generics companies to “freely produce medicines created by foreign drug companies at a fraction of the cost” [2]. Process patents, on the other hand, were recognized as they were seen as an incentive for domestic manufacturers to develop “cheaper methods of making expensive patented products”, and a way for the Indian government to keep drug prices low [4]. In 1995, India became a member of the World Trade Organization (WTO), and was compelled to revise its patent laws following a ten-year transition period [11]. India’s adjusted laws had to comply with the Trade-Related Aspects of Intellectual Property Rights (TRIPS) Agreement, the “WTO’s minimum standards for intellectual property protection” [4]. Thus, January 1st, 2005 saw the “implementation of substantially enhanced patent protection for pharmaceuticals” in India, in that drug products were now able to become patentable [12]. Following India’s commitment to the TRIPS Agreement, the *Times of India* accused the government of selling out to “rapacious” multinationals and making citizens pay for the sellout [13]. Many detractors argued that if India was fully compliant by 2006, as required under WTO obligations, changes in the legislation and regulation of pharmaceuticals would make India a net importer, instead of a net exporter in the sector. Others pointed out that the TRIPS Agreement is not consistent with the economic and social conditions of India and its terms are most harsh on those who are in the greatest need. Echoing this sentiment, a former president of the local pharmaceutical industry association noted that, “The (Patents) Act has taken into consideration the country’s socio-economic, developmental, technological, and public interest needs. Thanks to the Indian Patents Act … the prices of drugs … are reasonable and this has benefited the consumer at large” [14].

What is evident is that India has had a mixed approach towards the implementation of the TRIPS Agreement, availing itself to the full transition period for product patent protection, and delaying other commitments. For example, India was slow to set up a mechanism (known as the “mailbox” provision) that allows inventions to be notified to the patent officials, so that the invention can be established as “new”. It also stalled on a second required measure that involved exclusive marketing rights – that is if a state allows the new drug to be marketed, the firm that invented the pharmaceutical has the right to exclusively market the drug for a period of time. This strategy of delay suggests strongly that India, while committing to the spirit of the TRIPS Agreement, has also sought to ensure that its interpretation and implementation is in line with domestic preferences. India’s domestic patent provisions have been contested by the international research-based pharmaceutical industry (Table 1). For example, provisions in its domestic law that ban ‘evergreening’, a process in which minor reformulations to a preexisting drug can be used to extend patents (a common practice among pharmaceutical companies in developed countries); and second its criteria for ‘compulsory licensing’, a clause permissible under the TRIPS Agreement, which under extenuating circumstances, permits a country to “force a firm to license a patented drug to a generic company” [4,11].

**Table 1:** **International research-based pharmaceutical cases in India [**[3]**]**

**Company**	**Drug**	**Issue**	**Now**
Bayer	Nexavar (kidney cancer)	Patent office ordered Bayer to license its drug to an Indian firm to produce low-cost generics	IPAB rejected Bayer’s appeal to overturn compulsory license on Mar 4, 2013; Bayer to now appeal decision to Mumbai High Court
Bayer	Nexavar (kidney cancer)	Sued Cipla, an Indian firm, for patent infringement	Hearing in Dec 2012
Novartis	Glivec (leukaemia)	India refused to grant Swiss firm a patent in 2006	Indian Supreme Court rejected Novartis’ patent plea on April 1, 2013 after 7-year battle
Roche	Tarceva (cancer)	Sued Indian companies for infringing its patent	Delhi High Court dismissed Roche’s patent infringement suit in Sept 2012 after 4-year struggle
Roche	Valcyte (AIDS)	Patent office revoked Roche’s patent	Appeal pending to IPAB*
Gilead	Viread (HIV)	Patent office rejected two patents	Appealed; the case is still pending

*Intellectual Property Appellate Board.

The Indian Government has adopted a strategy to ensure that its global commitments do not undercut domestic priorities in the pharmaceutical system. That is, it is seeking to interpret its obligations under TRIPS in a manner that still permits the production of generic medicines and keeps medicine prices as low as possible to facilitate access to essential medicines [4]. In turn, this is presenting serious market challenges for the research-based pharmaceutical industry.

### The Novartis case

Glivec (imatinib mesylate), produced by the Swiss pharmaceutical giant Novartis, is used to treat Chronic Myeloid Leukemia (CML) and Gastrointestinal Stromal Tumours (GIST), and is patented in 35 countries across the world [11]. According to Lee [4], studies have shown that Glivec is “almost ten times more effective than traditional interferon therapy”, due to its ability to target specific cancer proteins. However, “the drug does not give a permanent cure from cancer … [it] only stalls its progress. For patients, the drug needs to be taken lifelong” [10]. For this reason, along with the fact that 95% of Indians do not possess private health insurance, its pricing plays a critical factor in cancer patients’ ability to access a continuous supply of Glivec for effective treatment. What is important to bear in mind, is that there is a significant price gap between the patented version of Glivec and its generic copy, as a monthly dose of the former can cost as much as USD$5,000 in the U.S., whereas a monthly dose of the latter can be purchased for just USD$200 in India [9]. In 2006, the Indian Patent Office rejected Novartis’ patent application for Glivec under Section 3(d) of the Indian Patents Act, stating that the drug was a modification of an existing substance, imatinib, and therefore represented a case of ‘evergreening’ [15]. Section 3(d) articulates that reformulations of pre-existing drugs, which do not improve the efficacy of the product, are ineligible for extended patents [16]. This provision was included primarily to safeguard public health interests [16]. Unfortunately, “neither the Indian patent statute nor its implementing rules define ‘efficacy’”, and there are no available guidelines for companies like Novartis seeking second-generation patents (i.e., extended patents on modifications of previous products) [16]. Thus, the interpretation of the word “efficacy” is central to this case. The Novartis case is a landmark case because it represents critical issues related to intellectual property protection and access to medicines, which will impact how multinational pharmaceutical companies conduct business in India in the future, as well as India’s role as the “Pharmacy of the Developing World”. India’s verdict is likely to serve as a model for other developing countries in terms of how they choose to interpret their obligations pursuant to the TRIPS Agreement [10].

### History of the case

**Figure 1:**
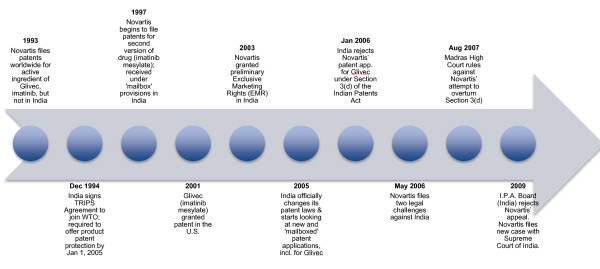
Timeline of the Novartis case.

Novartis’ attempts to patent Glivec in India span well over a decade (see Figure 1). In 1993, Novartis filed patents worldwide for imatinib, the precursor for the current version of its drug Glivec [11]. However, it did not do so in India as India at the time did not offer product patent protection [11]. In 1997, when Novartis developed the beta crystalline form of imatinib – imatinib mesylate – which it found to have 30% more bioavailability than its non-salt form (i.e., absorbed 30% more easily into the bloodstream), the company applied for a second round of patents, this time including India [11]. The patent application was received under India’s ‘mailbox’ provisions, a scheme which allowed companies to request patents while the Indian government transitioned towards a revised intellectual property legal system in 2005 at the behest of the World Trade Organization [4,11]. However, Indian generic producers were manufacturing and selling Glivec at less than 10% of the patented version’s price, compelling Novartis to put pressure on the Indian government to take a stance on intellectual property protection [4]. In response, the Indian government granted the company Exclusive Marketing Rights (EMR) until its application came up for review [4]. This decision put a stop to the majority of the production of generic versions of Glivec in India, thereby resulting in massive access barriers for individuals seeking affordable cancer treatment [8]. Several generic companies and not-for-profit organizations such as the Cancer Patients Aid Association (CPAA) rallied together to protest against Novartis’ EMR status, and filed an opposition against the company’s patent application, which was due for examination in 2005, the year when India would officially begin to look at both new and ‘mail-boxed’ patent requests [8]. In 2006, pursuant to Section 3(d) of the Indian Patent’s Act, the Indian Patents Office rejected Novartis’ patent application for its drug Glivec, citing that it did not demonstrate any significant changes in therapeutic effectiveness over its pre-existing form, which was already patented outside India [15]. In rebuttal, Novartis filed two legal challenges against the Indian government later that year – one appealing the rejection of its patent request, and the second contesting Section 3(d) of the Indian Patents Act, claiming that it did not comply with TRIPS, which India had ratified in 1994 [3]. In August 2007, the Madras High Court ruled against Novartis’s attempt to overturn Section 3(d), and in 2009, the Intellectual Property Appellate Board in India rejected the company’s appeal against the rejection of its patent application [2]. Novartis then filed a new case with the Indian Supreme Court, disputing the basis of these decisions, and the final decision came out in early April 2013.

## Methods

We searched for a wide range of sources, including scholarly journals, briefing documents, newspaper and magazine articles as well as video interviews with key stakeholders in order to gather a comprehensive and up-to-date view of the Novartis case in India and its far-reaching implications on the global pharmaceutical industry. Scholarly sources were peer-reviewed, and were drawn from numerous databases, primarily through ProQuest (Interdisciplinary) via the University of Toronto Libraries website. Our searches consisted of keywords such as ‘Novartis’, ‘India’, ‘intellectual property’, ‘patent’, and ‘Glivec’ to keep the perspective broad and view the issue from as many angles as possible. Sources were selected based on their relevance to the topic and date of publication from 1994 to 2013. Data sources were interpreted and analyzed according to our own prior knowledge and understanding of the exigencies of the TRIPS Agreement. Finally, our research was assembled into the current report to showcase the progression of the Novartis case over time and highlight its significance to intellectual property rights and access to medicines in the larger global health context.

## Results and discussion

### Novartis’ perspectives

According to Novartis, Section 3(d) of the Indian Patents Act should not have applied to Glivec at all. The company asserts that the initial patented form of the drug, imatinib, was only the first step in developing the current version, and could not be administered to patients [15]. Only by making the drug in its current salt form, imatinib mesylate, could it become a viable treatment [15]. Novartis scientists cite that this new form allows patients to take the drug “in a pill form that…deliver[s] consistent, safe, and effective levels of the medicine” [12]. Furthermore, imatinib mesylate exhibits 30% more bioavailability and is more stabile during production [6]. These improvements led to the awarding of a second-generation patent in the United States in 2001 [3]. Novartis also argued, that “Indian patent laws should distinguish between patented inventions and the version of the drug that is on the market for patients” [6]. The company sought a patent for the original molecule to protect the invention, however, a new patent was being sought to protect the medicine.

Moreover, not only did Novartis challenge India’s decision to reject its patent application for Glivec, but it also questioned the validity of Section 3(d) under the TRIPS Agreement. Citing Article 27 of TRIPS, which “generally mandates patentability where inventions are new, involve an inventive step (or are non-obvious), and are capable of industrial application (or are useful)” [5], Novartis claims that imatinib mesylate represented an “inventive step” in the drug development process due to its 30% increase in bioavailability [5]. To be sure, the TRIPS Agreement is sufficiently vague and does not explicitly define what an “inventive step” entails [5]. India technically has the flexibility to interpret criteria under TRIPS based on national socioeconomic conditions [5]. Novartis, on the other hand, refuted this position, and argued that lax patent laws like those in India may lead to the stifling of innovation in the pharmaceutical sector [16]. Novartis and other pharmaceutical companies argue that the research and development process is long and expensive, and a stable system that protects intellectual property rights is essential so that companies can recoup their expenses [16]. According to Novartis, access to the latest life-saving drugs for people in India and the developing world is dependent on patent protection [16]. In its efforts to safeguard public health interests by denying Glivec a patent, India may just as easily be compromising the very system that helps create new lifesaving medicines for the people who need them.

### Perspective of government of India

The Indian Government argued that Novartis’ patent application for Glivec should be rejected because the modified version of the drug did not exemplify a significant change in therapeutic effectiveness over its previous form [10]. It stated “… the selection of a salt of the active ingredient with the purpose of improving bioavailability (also referred to as ‘evergreening’) is well-known in pharmaceutical art” [10]. India’s patent laws contain certain provisions such as Section 3(d), which ban this sort of practice in order to protect access to medicines for its population [8]. The pricing of cancer treatment is arguably the most important factor in determining India’s position in the case: a monthly dose of the patented version of Glivec (around USD$2,600 per patient) is over three times an average Indian’s annual income.

India has also argued that under the Doha Declaration on the TRIPS Agreement and Public Health of 2001 its actions are legal. This provision states that the “the TRIPS Agreement can and should be interpreted and implemented in a manner supportive of WTO Members’ rights to protect public health and, in particular, to promote access to medicines for all” [11]. The Indian government and its supporters argue that Section 3(d) of the Indian Patents Act, although not explicitly contained within TRIPS, allows them the ability to interpret patent laws in favor of national public health interests [11]. Therefore, Indian patent laws are indeed constitutional, contrary to claims made by Novartis’ legal representatives.

### Implications of India’s Supreme Court rejection of Novartis’ appeal

The Novartis case started in 1998 when the company filed a patent application, which was denied in 2006, and only reached a final decision in April 2013 when India’s Supreme Court determined that the beta crystalline form of Glivec was ultimately not patentable. Section 3(d) of the Indian Patent Act, which expresses that minor changes to existing molecules will not be deemed as sufficient for further patent protection, was critical to this case [17]. Indeed, the court indicated that “therapeutic efficacy needs to be enhanced in order for an adapted compound to be considered to fall outside of the Section 3(d) exclusion” [17]. The verdict of the Novartis case “confirms the right of India’s Parliament to implement public health safeguards available under the TRIPS Agreement” [18].

Furthermore, the decision to reject Novartis’ patent “has global significance since India’s generic drug industry, valued at approximately USD $26 billion, supplies much of the cheap medicine used in the developing world” [19]. It illuminates how a government will take action to ensure that medicines are made affordable for its population. Also, this outcome may very well serve as an important model to other developing countries, which would want to ensure that their patent laws do not result in public health compromises. It is relevant then that both Argentina and Philippines adopted a law similar to Section 3(d) [20].

The 300,000 patients currently taking the drug and their advocates welcomed the verdict [21]. According to Dr Unni Karunakara, the Médecins Sans Frontières (MSF) International President: “The Supreme Court’s decision now makes patents on the medicines that we desperately need less likely. This sends a very strong signal to Novartis and other multinational pharmaceutical companies that they cannot try to game Indian patent law” [22]. This decision has “no precedent”, explained Pratibha Singh, a lawyer from the Indian drug manufacturer Cipla, because from now on “patents will be given for genuine inventions, and repetitive patents will not be given for minor tweaks to existing drugs” [19].

Novartis’ reaction was not surprisingly stated as an economic and research threat. It stated that the “decision … discourages innovative drug discovery essential to advancing medical science for patients” [20]. It further stated that: “Novartis most certainly continues to seek patents for its innovative products in India … but will be cautious in investing in India especially with regard to introduction of innovative medicines” [20]. However, Novartis in an effort to minimize negative publicity also strategically noted that the 16,000 people (which represent around 95% of the patients currently taking the branded drug Glivec in India) from the “Novartis Glivec International Patient Assistance Program” will continue to receive the drug free of charge. [22].

## Conclusion

The Novartis case arguably sets an important precedent for access to medicines by putting the pharmaceutical industry on the reach of patent law. The Supreme Court of India’s decision may very well serve as a future model for other developing countries in how they choose to interpret and implement the TRIPS Agreement. This case illuminates how India is respecting its global obligations concerning intellectual property laws while ensuring that domestic needs are respected by interpreting its legal obligations in a way that is commensurate with domestic preferences and needs. The ruling puts social justice over commercial interests and also helps India’s own domestic industry. This is the first time that Indian law has been implemented to prohibit patents on drugs with only minor changes to an existing one. Now, only truly new and innovative medicines with real therapeutic impact will be protected via patenting. What we see in the case of India is a complex game that results in tension between global trade commitments and domestic public health concerns. The latter in this case has clearly taken precedence.

## Abbreviations

CML: Chronic myeloid leukemia; CPAA: Cancer Patients Aid Association; EMR: Exclusive marketing rights; GIST: Gastrointestinal stromal tumors; MSF: Médecins sans frontières; TRIPS: Trade related aspects of intellectual property rights; WTO: World Trade Organization.

## Competing interests

The authors declare that they have no competing interests.

## Authors’ contributions

RG developed the research question, undertook analysis, writing and editing. JCK advised on the research question and undertook research, analysis, writing and editing. Both authors read and approved the final manuscript.

## Authors’ information

RG is a Masters in Public Health student at the Dalla Lana School of Public Health at the University of Toronto.

JCK is an Associate Professor and Director of Global Health at the Leslie Dan Faculty of Pharmacy and Munk School of Global Affairs at the University of Toronto.
